# Metabolic interactions between *Toxoplasma gondii* and its host

**DOI:** 10.12688/f1000research.16021.1

**Published:** 2018-10-30

**Authors:** Martin Blume, Frank Seeber

**Affiliations:** 1 NG2 - Metabolism of Microbial Pathogens, Robert Koch-Institute, Berlin, Germany; 2 FG16 - Mycotic and Parasitic Agents and Mycobacteria, Robert Koch-Institute, Berlin, Germany

**Keywords:** Metabolism, intracellular parasite, metabolomics, bradyzoite

## Abstract

*Toxoplasma gondii *is an obligate intracellular parasite belonging to the phylum Apicomplexa that infects all warm-blooded animals, including humans. *T. gondii* can replicate in every nucleated host cell by orchestrating metabolic interactions to derive crucial nutrients. In this review, we summarize the current status of known metabolic interactions of *T. gondii *with its host cell and discuss open questions and promising experimental approaches that will allow further dissection of the host–parasite interface and discovery of ways to efficiently target both tachyzoite and bradyzoite forms of *T. gondii*, which are associated with acute and chronic infection, respectively.

## Introduction

*Toxoplasma gondii* is an obligate intracellular parasite that infects all warm-blooded animals, including birds and humans, where it can replicate in all nucleated cells within a non-fusogenic compartment termed the parasitophorous vacuole (PV). The fast-replicating tachyzoites cause the acute phase of the infection. However, in response to immune pressure, differentiation into the slow-growing or quiescent bradyzoites, which is accompanied by the transformation of the PV membrane (PVM) into a cyst wall, marks the onset of the chronic phase. In general, *T. gondii* transmission to humans occurs via ingestion of tissue cysts contained in contaminated undercooked meat products. In addition, humans can be infected following exposure to oocysts shed by the definitive feline host via contaminated food or water. *T. gondii* infections are largely asymptomatic during both the acute and chronic phases, and the chronic stage persists for the life of the host. However, upon severe immunosuppression, rapid replication of *T. gondii* tachyzoites, derived from the reactivation of encysted bradyzoites, can cause severe symptoms such as encephalitis, which is potentially fatal. Additionally, primary infection during pregnancy is associated with serious consequences for newborns, ranging from blindness and deafness to mental retardation and stillbirth. However, as is the case with many other intracellular pathogens, the intruder is not unnoticed, as evidenced by transcriptomic and metabolomic disturbances within the host. The latter uses these cues to counteract the infection on biochemical as well as immunological levels (for review, see 1– 3).

Both tachyzoites in PVs and bradyzoites in cysts integrate tightly with their host cells to ensure nutrient supply for optimal proliferation and persistence, which define the characteristics of the specific life cycle stage ^4– 6^. In this review, we will briefly outline recent findings of how *T. gondii* tachyzoites and bradyzoites interact with their host cells for the acquisition of essential nutrients. Recent progress in molecular genetics as well as biochemical and metabolomic methods has resulted in a better understanding of the competition for the shared pool of nutrients. In many respects, *T. gondii* exhibits metabolic traits similar to those of *Plasmodium* species, the causative agents of malaria, particularly with regard to the understudied hepatic stage. Therefore, insights from this pathogen’s metabolism can also instruct efforts to exploit metabolic dependencies as drug targets in more than one apicomplexan parasite.

## Nutrient acquisition from the host cell: not only a transporter issue

*T. gondii* tachyzoites and bradyzoites replicate intracellularly and therefore need to acquire nutrients from their host cells. The parasite establishes a vacuole that is initially composed of host lipids but during its active invasion process excludes most host proteins ^7^. The PVM is then heavily modified by parasite proteins that mediate protein export and the import of lipidic and polar metabolites. It has long been known that the PVM is freely permeable for molecules as large as 1,300 Da ^8^, but only recently were two parasite proteins—GRA17 and GRA24—defined as the molecular constituents of this pore ^9^. It is thought to be permissive for non-directional passive transport of small nutrients, such as vitamins, sugars, amino acids, nucleobases, nucleosides, and nucleotides. It provides a putative mechanism for the export of catabolites, such as lactic acid and, to a lesser extent, alanine and bicarbonate ^10^. Besides this pore, an intravacuolar membranous tubulo-vesicular network (membranous tubules and vesicles that are bridging the PVM with the parasite ^11^) has been shown to be involved in the uptake of host proteins and lipids ^12– 14^. Whether other host metabolites can enter the PV via this route and likewise whether it is used on the other hand by the parasite as a “waste pipe” are unknown.

**Figure 1.**
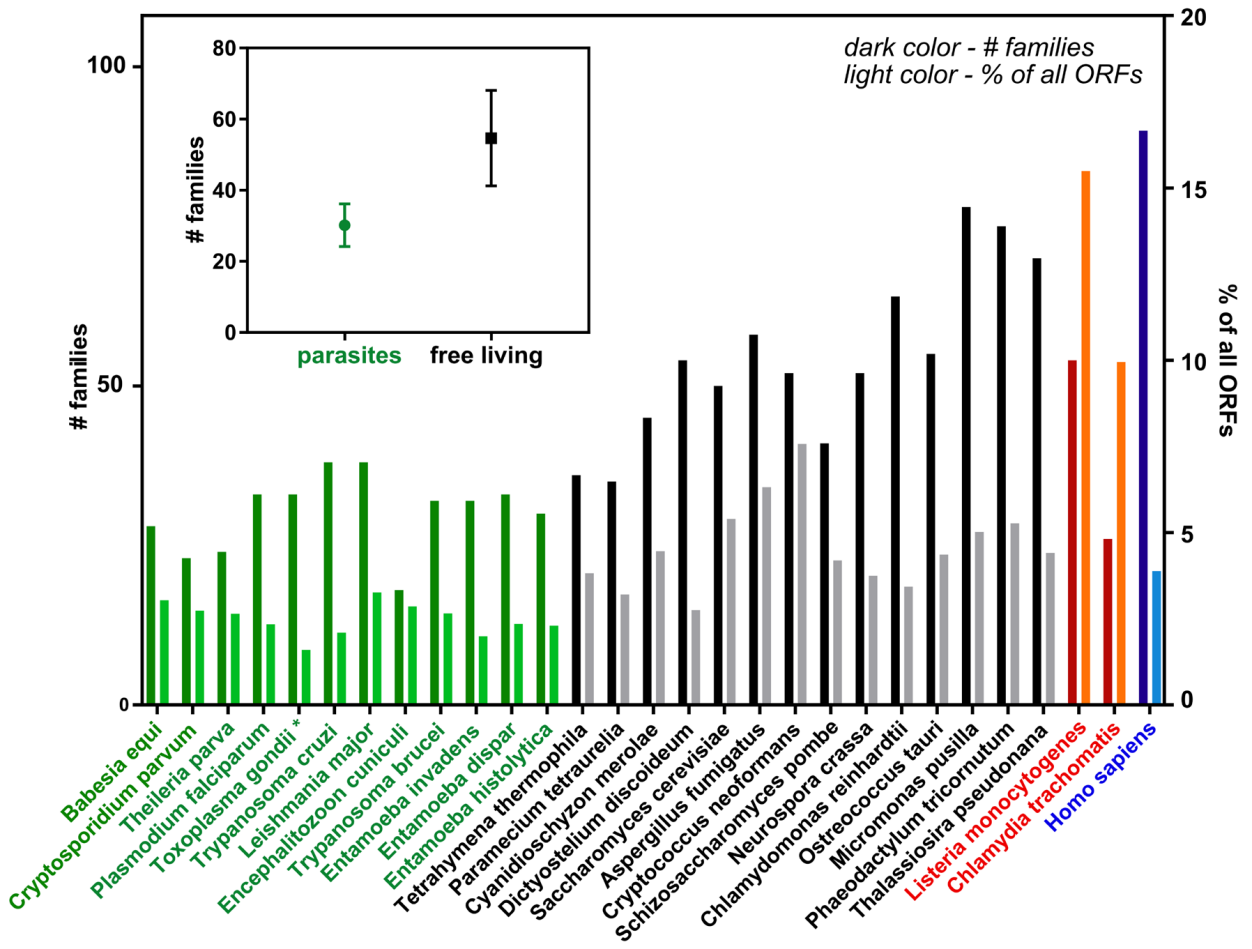
Quantitative comparison of computationally annotated transporters between parasitic and non-parasitic unicellular organisms. Data were extracted from TransportDB 2.0 ^20^. Dark-colored bars represent the numbers of different transporter families per given genome, whereas the light-colored bars give the percentage of all predicted transporter proteins per entirety of open reading frames (ORFs). Green indicates parasitic organisms and black indicates free-living organisms. *Data are from 15 since *Toxoplasma gondii* is absent in the current release of TransportDB 2.0. The inlet provides the mean ± standard deviation of the number of families of both groups. For comparison, intracellular bacteria (red) and humans (blue) are also shown.

Most polar metabolites are imported through a range of transporters in both the PVM and the parasite’s plasma membrane. To date, a small subset has been functionally characterized; hence, there likely exists a larger interaction surface and undiscovered redundancy. Strikingly, however, there are lower numbers of computationally annotated transporter families in the genomes of parasitic protozoa ^15^ when comparing both intracellular and extracellular living parasites with unicellular free living organisms ( Figure 1). This is despite the need to scavenge as many nutrients as possible from the environment. Explanations for these lower numbers could include a broader substrate specificity of individual transporters combined with a minimal need for diversification due to host niches with complex but predictable compositions. Several recent studies have started to shed light on the importance of transporter families and channels for parasite survival. Consequently, these molecular entities constitute potential drug targets, some of which are currently being exploited ^16^.

## Ingredients to make a tachyzoite

**Figure 2.**
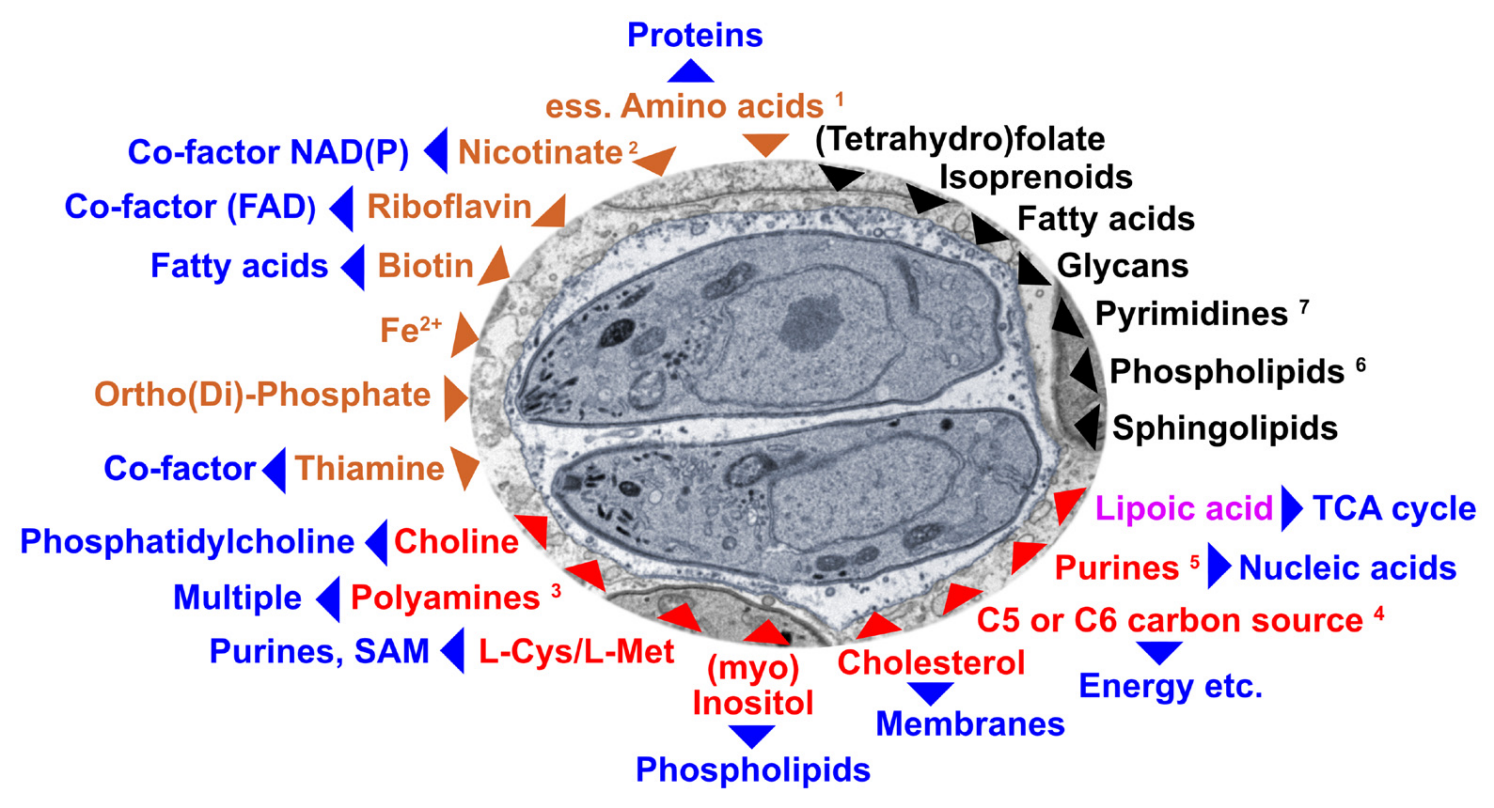
Currently known or predicted dependencies of *Toxoplasma gondii* tachyzoites on host cell–derived metabolites. Based on data from 4, 18. Two *T. gondii* tachyzoites within a parasitophorous vacuole (light blue) are shown within a fibroblast host cell (gray). Red arrowheads indicate uptake of compounds that can be synthesized by mammalian cells, whereas brown arrowheads mark uptake of those substances for which both organisms are auxotrophic and thus they compete. Metabolites in black are scavenged when available but are not strictly essential whereas the pathways they are involved in are. Blue arrowheads denote the end products for which the imported metabolites are precursors or essential co-factors (or both) for their synthesis. Lipoic acid (pink) is special since it is synthesized in one compartment of the parasite (apicoplast) but apparently unable to reach another organelle (mitochondrion) where it is also required; thus, it has to be scavenged from the host ^32^. ^1^L-Arg; L-Trp; L-His; L-Lys (or L-2-aminoadipate 6-semialdehyde); L-Ile (or (S)-3-methyl-2-oxopentanoate); L-Val (or 3-methyl-2-oxobutanoate); L-Leu (or 4-methyl-2-oxo-pentanoate); L-Phe (or phenylpyruvate). ^2^Nicotinate or nicotinate-D-ribonucleoside or nicotinamide. ^3^Putrescine, spermine, ornithine. ^4^D-fructose or D-glucosamine or D-glucose or D-mannose or D-ribose or D-sorbitol or 2-Deoxy-D-ribose. ^5^Adenine or adenosine or guanine or guanosine or inosine or hypoxanthine or xanthine. ^6^Phosphatidylserine, phosphatidylcholine, phosphatidylethanolamine, phosphatidic acid. ^7^Uracil, uridine, cytidine, deoxyuridine, deoxycytidine.

Global genome-based models of *T. gondii* metabolism can provide an estimated minimal set of required nutrients. These flux balance models (FBMs) use gene annotations to predict the presence of metabolic pathways and use estimated parasite biomass composition and ATP consumption rates to model corresponding fluxes. To date, two FBMs of *T. gondii* metabolism have been generated and published: iCS382 ^17^ and ToxoNet1 ^18^. The recent, more comprehensive study by Tymoshenko *et al*. resulted in a proposed minimal set of required nutrients for tachyzoite growth that are illustrated in Figure 2 ^18^. It should be emphasized that validation of nutrient dependencies is no easy feat for intracellular parasites, as they are in constant competition with the host and no apicomplexan parasite has yet been reported to replicate indefinitely in axenic media. In addition nutrients are conditionally dependent on the presence of other nutrients and environmental conditions.

In accordance with ToxoNet1 predictions, tachyzoite growth depends on successful competition for a number of co-factors and vitamins with the host cell. These small molecules fulfill vital metabolic functions in the parasite ( Figure 2). We linked these nutrient-dependent pathways with the set of essential metabolic pathways as identified through FBM modelling ^18^ and assigned the respective “phenotype scores” of the genome-wide CRISPR (clustered regularly interspaced short palindromic repeats) screen of Sidik *et al*. ^19^ to the respective enzymes ( Table S1). As expected, these pathways contain mostly essential genes with exceptions such as putative phosphatidylinositol synthetase, NAD ^+^ synthetase, and a sphingolipid desaturase, a loss of which do not cause significant fitness penalties ^19^. This may indicate that *T. gondii* is able to scavenge products of these enzymes in some form; it might also reflect incorrect gene annotations or insufficient time elapsed between gene knockout and expression of a fitness defect in those experiments ^19^.

The major carbon sources of *T. gondii* are glucose and glutamine ^21^. In addition, the parasite can use exogenous acetate to elongate fatty acids if available ^10, 22^.

Amino acid auxotrophies of *T. gondii* tachyzoites have been a long recurring subject of research but, to our knowledge, have not been systematically and comprehensively tested. As is true for many unicellular pathogens, *T. gondii* requires exogenous tryptophan ^23, 24^. It is also auxotroph for arginine ^25^ and tyrosine ^26^. Besides glutamine, a range of amino acids are imported, as shown recently by gas chromatography–coupled mass spectrometry (GC/MS). These include alanine, valine, leucine, isoleucine, proline, glycine, serine, threonine, methionine, and tyrosine ^24, 27^. Phenylalanine import ^28^ was observed by using Raman spectroscopy. Complementarily, *T. gondii* has been shown to be able to synthesize alanine, glutamine, and aspartate by stable isotope-resolved GC/MS ^10^. Recently, several amino acid transporters have been identified as part of a transporter family that appears to be specific to apicomplexans ^24^. Arginine and tyrosine transport activity has been assigned to *T. gondii* apicomplexan amino acid transporter 1 and 5-3 (TgApiAT1 and TgApiAT5-3), respectively ^24, 27, 29^. Other amino acids such as histidine, lysine, branched-chain amino acids, and cysteine or methionine have been considered to be essential ^18^ but this remains to be experimentally verified.

In addition to proteinogenic amino acids, *T. gondii* tachyzoites are thought to rely on uptake of ornithine and spermine to reverse-synthesize polyamines ^30^. The metabolic roles that are fulfilled by these compounds in *T. gondii* are still ill defined ^30, 31^.

Besides amino acids, purines are a major nutrient for *T. gondii* and required for nucleic acid synthesis (reviewed in 33). As a purine auxotroph, *T. gondii* imports purines in various forms through three transporters. TgAT1 is a low-affinity nucleoside transporter for adenosine and inosine ^34^. Furthermore, the existence of two uncloned transporters has been postulated by detailed radiolabeling experiments. TgNBT1 is a high-affinity purine base transporter that accepts hypoxanthine, xanthine, and guanine as substrates. TgAT2 is a broad-spectrum nucleoside transporter ^35^.

*T. gondii* imports a number of vitamins that are needed as co-factors for essential enzymes.

Biotin is a co-factor for several carboxylase reactions, including the acetyl-CoA carboxylase (ACCase), which is essential for *de novo* fatty acid synthesis in the apicoplast (FAS2), a relict plastid ^19, 36, 37^ ( Table S1). Biotin was not mentioned as an essential co-factor in previous lists, but *T. gondii* appears to lack the canonical biotin synthesis pathway ^38^. It is readily taken up through the host cell as indicated by its use for tagging proteins via a promiscuous ubiquitin ligase BirA ^39^. The qualification of biotin as an essential vitamin for *T. gondii* is supported by recent data showing that a biotin ligase of *Plasmodium falciparum*, responsible for post-translational modification of its ACCase, is required for the development of liver stages ^40^. Accordingly, we added the respective homolog of *T. gondii* to the list of essential genes in Table S1.

Folates take part in the synthesis of pyrimidines and amino acids and presumably are taken up through the putative TgBT1 transporter ^41^ and are also synthesized (reviewed in 33).

Thiamine is a co-factor for transketolases and dehydrogenases that act on pyruvate, alpha-ketoglutarate, and branched-chain keto amino acids ^42^. To date, there is no biochemical evidence of thiamine uptake. Interestingly, *T. gondii* (M. Blume, unpublished data) and the related apicomplexan *P. falciparum* ^43^ are sensitive to the analog oxythiamine, indicating that this co-factor can be salvaged and is incorporated into enzymes.

Similarly, lipoic acid is an essential co-factor for a number of dehydrogenases and is being scavenged from the host cell in spite of being synthesized by the parasite in the apicoplast ^32^ ( Figure 2).

In contrast to these co-factors, tachyzoites appear unable to scavenge pantothenate, a precursor for coenzyme A, in significant amounts. Instead, they possess the entire synthesis machinery for pantothenate ^44^ and parasite growth is sensitive to its pharmacological inhibition.

The uptake of nicotinate or nicotinamide and riboflavin has been proposed to be required for growth of *T. gondii* ^18^; however, to date, no biochemical evidence has been reported. Not surprisingly, FBMs also predict that *T. gondii* requires inorganic ions such as iron ^45, 46^ and phosphates. Also, their uptake mechanisms remain unknown.

In addition to importing small molecules that act as building blocks, *T. gondii* imports proteins from the cytosol of its host cell and digests them in its endolysosomal system via cathepsin L and other proteases ^12^. The underlying uptake mechanism is unclear but may involve the intravacuolar network of tubular membranes as a potential delivery route. Interestingly, a transient endocytic structure in the parasite plasma membrane that can be triggered by excess supply of oleic acid ^13^ has been implicated in lipid droplet uptake from the host and may also be responsible for import of host proteins.

## Lipid uptake: the dose is the poison

To sustain its rapid replication, *T. gondii* tachyzoites need to synthesize large amounts of lipids and import precursors. Exciting new research has established that host lipid droplets present a mobilizable source of neutral lipids and fatty acids for tachyzoites. Infected cells exhibit an increased abundance of lipid droplets ^47, 48^ which can be further increased by exogenous oleic acid ^13^. Under these conditions, *T. gondii* scavenges oleic acid or neutral lipids (or both) from Rab7-positive host lipid droplets and stores excess lipids in deposits as triacylglycerides in its cytosol. The disruption of this storage pathway has been shown to be detrimental to tachyzoites and bradyzoites *in vitro* ^13, 49^. Under normal culture conditions, fatty acid import results in an even cellular distribution of the acquired fatty acids and confers enhanced growth ^50^. Interestingly, the fusion of host mitochondria around the PV has been shown to limit access of *T. gondii* to host fatty acids and decrease its replication rate ^50^. These studies report different intracellular distributions of internalized fatty acids that indicate distinct fates of host lipid droplet content. However, it is important to note that the employed fluorophore-linked lipids such as C4-BODIPY-C9 or BODIPY-FL-C12, though conceptually attractive, differ substantially in their biophysical properties from the lipids of interest. These include altered flip-flop rate and transfer between organelles compared with their parental lipids ^51^. Regardless, these studies suggest that the parasite undertakes largely unregulated import of lipids and depends on control mechanisms from its host cell instead. There appears to be a need to fine-tune the fatty acid supply of *T. gondii* since fatty acid scavenging is supportive of growth only in low doses ^50^ and becomes harmful at higher concentrations ^13^. Shielding parasites from fatty acid uptake by enwrapping their PV in host mitochondria appears to be a defense mechanism ^50^. It will be important to see how general this proposed mechanism is operating since, in the low virulence type II strains, the host mitochondria do not associate with the PVM ^52^.

The uptake of fatty acids is complemented by *T. gondii*’s comprehensive capabilities to produce fatty acids on its own. GC/MS-based methods revealed that the parasite can synthesize myristic and palmitic acid *de novo* via the FAS2 pathway in the apicoplast and also possesses a set of elongases and the FAS1 pathway ^53^. In addition, GC/MS-resolved stable isotope labelling patterns revealed salvage of unsaturated long chain and very long chain fatty acids from its host ^54^.

Other precursors for major lipids include choline, which is needed for phosphatidylcholine synthesis ^55^, and this pathway can be poisoned with the choline analog di-methylethanolamine ^56^. Phosphatidylinositol is part of the lipidome of *T. gondii* but its synthase appears non-essential ( Table S1). Also, its precursor myo-inositol is not synthesized from glucose ^10^, indicating that *T. gondii* can access exogenous inositol and may also import this phospholipid. In addition, *T. gondii* acquires several other lipids directly from its host. These include low-density lipoprotein-derived host cholesterol for which the parasite is auxotroph (reviewed in 57) and sphingolipids from Golgi-derived vesicles ^55, 58^.

Another important class of lipidic molecules are isoprenoids. These are implicated in a number of essential processes that include signaling, trafficking, energy metabolism, and protein translation (reviewed in 59). *T. gondii* synthesizes the precursors isopentenyl pyrophosphate and dimethylallyl pyrophosphate in its apicoplast via the non-mevalonate pathway ^60^. These molecules are the building blocks for terpenoids via a multifunctional farnesyl diphosphate synthase (TgFPPS) ^61^. Interestingly, *T. gondii* also scavenges products of this enzyme, including farnesyl- and geranylgeranyl-pyrophosphates from its host, making TgFPPS knockout mutants dependent on corresponding host isoprenoid synthesis ^62^.

## The state of bradyzoite–host interactions: initial insights into a black box

Owing to their ease in cultivation, tachyzoites are clearly the most investigated stage of *T. gondii*. However, the vast majority of *T. gondii*’s lifetime in intermediate hosts is spent in the bradyzoite stage. Much less is known about how these stages interact with their host cell and which metabolic factors permit persistence. *T. gondii* tissue cysts are frequently found in brain, muscle, eye, and cardiac tissue ^63^. It is unclear whether the parasite displays tropism toward particular cell types or whether some are more permissive for long-term residence. Interestingly, *in vitro* parasites display elevated spontaneous stage conversions in cell cycle–arrested fibroblasts ^64^ and terminally differentiated skeletal muscle cells ^65^. This suggests that non-proliferating cells express an as-yet-unknown effector or provide a generally stressful environment. In this respect, it is noteworthy that proliferating cells maintain a glycolytic metabolic profile, with large pools of glycolytic intermediates and high rates of lactic acid production, to support their biosynthetic activities ^66^. This environment may equally favor the biosynthetic activity of growing *T. gondii* tachyzoites. Consistently, host cells that produce high levels of lactic acid promote tachyzoite growth over bradyzoite formation ^67^. The induction of bradyzoites *in vitro* is considered to work by slowing down tachyzoite growth via a range of stressors, such as limitation of arginine and bicarbonate as well as basic and acidic pH stress ^63^. The resulting encysted bradyzoites adopt a spectrum of cell division rates ^68^ that may introduce a degree of flexibility in their need for pyrimidines for nucleic acid synthesis and consequently contribute to their resistance to antifolates such as pyrimethamine. However, bradyzoites appear sensitive to inhibition of nutrient storage and turnover pathways including autophagy ^69^, lipid storage ^70^, and carbohydrate storage ^71^. These findings may reflect the fact that *T. gondii* deals with oversupply of nutrients by storage rather than by regulating respective import pathways. In particular, slowly dividing bradyzoites appear to depend on the integrity of the regulation of these storage pathways to avoid detrimental accumulation of material.

## Future methods to study host–parasite interactions

As outlined above, comprehensive metabolomic approaches were instrumental in defining metabolic interactions ^72^ as well as synthesis and import capabilities of fatty acids and amino acids in tachyzoites ^10, 24^. A similar approach with bradyzoites requires the development of efficient methods for the *in vitro* culture and purification of tissue cysts in their natural metabolic state. Strategic introduction of stable isotopes into parasite polymers such as DNA and proteins might be helpful to measure the activity of metabolic pathways and decrease the number of cysts needed ^73^.

Comprehensive liquid chromatography–coupled mass spectrometry (LC/MS)–based metabolomic approaches will also help to define which amino acid synthesis pathways are active in *T. gondii*. By measuring the levels and turnover of intermediates directly, metabolomics can bridge the gap that exists between annotated gene sets and a functional pathway. Such associations are very indirect since function will depend on regulatory mechanisms at the transcriptomic, proteomic, and metabolomic levels ^74^. In addition, the accuracy of functional predictions based on homology are limited. For instance, a putative lysine decarboxylase implicated in lysine degradation has been shown to act as a glutamate decarboxylase within the GABA shunt instead ^10^.

Similar limitations apply to conclusions based on transcriptomic and proteomic data. While these approaches are powerful to functionally associate genes and resolve differences of physiological states of the parasite under different conditions, such as host environments ^64^ and stage conversion states ^75, 76^, functional implications on the phenotype level and mechanistic insights are constrained by functional gene annotations. Combined multi-omics approaches might provide insights beyond the metabolic dimension of host–parasite interactions in the future ^77^.

In addition to the use of metabolomic techniques to study parasite–host interactions, the use of more sophisticated cellular systems such as neurons and myotubes, organoids ^77^, or mice ^78^ holds great potential. This is illustrated by the fact that *T. gondii* and coccidian-specific genes are the least fitness-conferring ones for the survival in fibroblasts ^19^.

In conclusion, there remain many open questions about how parasite and host metabolism interact, in particular with respect to dormant bradyzoites that are key for transmission and global prevalence of *T. gondii*. Future metabolomic approaches will be instrumental in further dissecting the host–parasite interface and discovering ways to efficiently target both tachyzoite and bradyzoite forms of *T. gondii*.

## Abbreviations

ACCase, acetyl-CoA carboxylase; FAS2, fatty acid synthesis in the apicoplast; FBM, flux balance model; GC/MS, gas chromatography-coupled mass spectrometry; PV, parasitophorous vacuole; PVM, parasitophorous vacuole membrane; TgFPPS, farnesyl diphosphate synthase from *Toxoplasma gondii*

## Supplementary material

**Supplementary File 1.** Table S1. Essential metabolic pathways and associated genes based on FBM Toxonet1.
